# SMPD3 as a Potential Biomarker and Therapeutic Target in Hepatocellular Carcinoma

**DOI:** 10.1155/ijog/5443244

**Published:** 2025-03-11

**Authors:** Dan Zhu, Lei Cao

**Affiliations:** Department of Hepatobiliary and Pancreatic Surgery, Harbin Medical University Cancer Hospital, Harbin, Heilongjiang, China

**Keywords:** cell proliferation, hepatocellular carcinoma, prognosis, Sphingomyelin phosphodiesterase 3, survival analysis

## Abstract

**Background and Aims:** Hepatocellular carcinoma (HCC) is a prevalent and aggressive liver cancer with high mortality rates. Sphingomyelin phosphodiesterase 3 (SMPD3) has recently been suggested to play an antitumor role in several cancers. This study is aimed at investigating the role of SMPD3 in HCC and its potential as a prognostic marker and therapeutic target.

**Methods:** A retrospective cohort study of HCC patients was conducted using clinical data from our hospital. Survival analyses, including Kaplan–Meier and multivariate Cox regression, were performed to assess the impact of SMPD3 expression on survival. Further analyses were carried out using data from The Cancer Genome Atlas (TCGA) HCC cohort. In vitro and in vivo experiments were conducted to evaluate the effects of SMPD3 overexpression on HCC cell lines and tumor growth in mice.

**Results:** High SMPD3 expression level was associated with improved survival in both our cohort and TCGA cohort. Multivariate Cox regression analysis confirmed high SMPD3 expression level as an independent predictor of better survival outcomes. In vitro and in vivo experiments demonstrated that SMPD3 overexpression significantly decreased HCC cell proliferation, migration, and invasion and inhibited tumor growth in a nude mouse model.

**Conclusions:** SMPD3 plays a protective role in HCC by inhibiting tumor growth and progression. Its high expression is associated with better survival outcomes and may serve as a promising prognostic marker and potential therapeutic target in HCC. Further research into the molecular mechanisms of SMPD3's antitumor effects could lead to novel therapeutic strategies for HCC.

## 1. Introduction

Hepatocellular carcinoma (HCC) is the most prevalent form of liver cancer, constituting approximately 75% of primary liver malignancies globally [1, 2]. HCC arises from chronic liver disease and cirrhosis, often linked to factors such as Hepatitis B or C virus infection, alcoholic liver disease, and nonalcoholic fatty liver disease [3, 4]. The incidence of HCC continues to increase worldwide, making it a significant public health concern. While advancements in early detection and treatment options, including surgical resection, liver transplantation, and localized therapies, have emerged [5], HCC remains associated with high mortality rates due to its aggressive nature and late-stage diagnosis [6].

The exploration of molecular and cellular mechanisms underlying HCC has opened new avenues for therapeutic interventions. Among these, the sphingolipid metabolic pathway has gained attention due to its involvement in cell proliferation, apoptosis, and differentiation [7–10]. Sphingomyelin phosphodiesterase 3 (SMPD3), also known as neutral sphingomyelinase-2 (nSMase2), is an enzyme that catalyzes the hydrolysis of sphingomyelin to produce ceramide and phosphocholine [11–13]. Ceramide, the bioactive product of SMPD3, plays a pivotal role in regulating cellular stress responses and has been implicated in tumor suppression [14–16].

In the context of HCC, the antitumor effects of SMPD3 are not fully understood and warrant further investigation. Recent research has suggested that SMPD3 might have a role in modulating cell cycle regulation, inducing apoptosis, and inhibiting cancer cell proliferation [17–19]. Additionally, the production of ceramide by SMPD3 could be a key pathway in mediating its antitumor activities [20–22].

This study investigates the antitumor role of SMPD3 in HCC. We aim to understand how SMPD3 expression influences HCC cell proliferation, apoptosis, and tumor growth. Using various in vitro and in vivo models, we seek to elucidate the molecular mechanisms through which SMPD3 exerts its antitumor effects and assess its potential as a therapeutic target in HCC. Our research aspires to contribute to the development of innovative treatment strategies that can enhance the prognosis and survival rates of patients with HCC.

## 2. Methods

### 2.1. Cohort Enrollment

A retrospective cohort study was conducted using patients diagnosed with HCC at our hospital. Patients with histologically confirmed HCC and complete clinical data were included in the study. Clinicopathological characteristics such as age, sex, tumor size, alpha-fetoprotein (AFP) levels, TNM (tumor, node, metastasis) stage, HBV infection status, differentiation, pathological grade, and resection margin status were collected for each patient. Informed consent was obtained from all participants, and the study was approved by the ethics committee of Harbin Medical University Cancer Hospital (No. SYZ23-312).

### 2.2. Immunohistochemistry (IHC) Staining

IHC staining was used to evaluate SMPD3 expression in HCC tissue samples. Tissue sections were first deparaffinized and rehydrated, followed by antigen retrieval using citrate buffer (pH 6.0) heated in a microwave for 20 min. To block endogenous peroxidase activity, sections were treated with 3% hydrogen peroxide for 10 min. The sections were then incubated overnight at 4°C with a primary antibody against SMPD3 (dilution 1:200). After rinsing, sections were treated with a biotinylated secondary antibody and then a streptavidin-HRP (horseradish peroxidase) complex. Detection was carried out using a 3,3⁣′-diaminobenzidine (DAB) substrate kit, and sections were counterstained with hematoxylin. Two independent pathologists evaluated the staining intensity and the percentage of positive cells to ensure reproducibility and accuracy.

### 2.3. Online Dataset

In addition to our hospital cohort, data from The Cancer Genome Atlas (TCGA) HCC cohort were utilized for further analyses. This publicly available dataset provided detailed information on gene expression profiles, clinical outcomes, and other relevant patient characteristics.

### 2.4. Cell Line

Human nontumorous hepatocyte cell line (L02) and four human HCC cell lines (HepG2, PLC/PRF/5, PLC/PRF/8, and Huh7) were used in this study. All cell lines were obtained from the Institute of Biochemistry and Cell Biology of the Chinese Academy of Sciences. Cells were cultured in regular Dulbecco's modified Eagle medium (DMEM) supplemented with 10% fetal bovine serum and 1% penicillin/streptomycin under 5% CO_2_ in a 37° incubator. The pcDNA3.0 plasmids were introduced to overexpress SMPD3 using vector plasmids as the control. The overexpression efficiency was tested by reverse transcription quantitative polymerase chain reaction (RT-qPCR) and Western blot assays.

### 2.5. Western Blot

Total protein was extracted from cells using radioimmunoprecipitation assay (RIPA) lysis buffer supplemented with protease inhibitors. Approximately 30 *μ*g of total protein was subjected to Western blot. Briefly, proteins were separated using 10% sodium dodecyl sulfate–polyacrylamide gel electrophoresis (SDS-PAGE) and electrotransferred onto polyvinylidene difluoride membranes (PVDF) (Millipore). Membranes were blocked with 5% skim milk and then incubated with corresponding primary antibodies. Then, the PVDF membrane was further incubated with secondary antibodies and subjected to film development to evaluate the immunoreactivity.

### 2.6. Proliferation Assay

Cell Counting Kit 8 (CCK8) (Dojindo) was used to assess cell proliferation capacities [23]. About 3000 transfected cells in 200 *μ*L DMEM were seeded in a 96-well plate per well, and measurements were conducted after 1, 2, 3, 4, and 5 days. At each time point, cells were incubated with 100 *μ*L of the reaction mixture (10 *μ*L CCK8 and 90 *μ*L DMEM) for 2 h and measured at a wavelength of 450 nm according to the manufacturer's protocol. Experiments were performed in triplicate.

### 2.7. Migration Assay

A wound-healing strategy was used to test the cell migration ability. Briefly, transfected cells were cultured to full confluence in six-well plates and scratched using 200 *μ*L sterile pipette tips. After scratching, the wells were gently washed with DMEM to remove the detached cells and cultured for 24 h. The six-well plates were then photographed under a light microscope to calculate the relative width of the scratched area. Experiments were performed in triplicate.

### 2.8. Invasion Assay

The Matrigel transwell method was used to assess the invasion capacities of stable transfected cells [24]. After precoating the transwells with Matrigel (1.0 *μ*g/mm^2^), 2 × 10^4^ cells were added into the upper chamber and cultured for 48 h. Invaded cells underneath the transwell membrane were fixed, stained, and counted under a light microscope. Experiments were performed in triplicate.

### 2.9. Xenograft Assay

The BALB/c nude mice were obtained from the Shanghai Institute of Materia Medica. Xenograft experiments were approved by the Medical Experimental Animal Care Commission of Harbin Medical University Cancer Hospital following the welfare of laboratory animals (No. SYZD23-084). Briefly, stable transfected PLC/PRF/8 cells were subcutaneously injected into male BALB/c nude mice at 5 weeks old. Seven days after injection, the subcutaneous tumor axis was measured every 3 days using a vernier. The tumor size was calculated as size volume = (*π* × length × width × width)/6. At 25 days after cell injection, all enrolled mice were sacrificed to isolate and weigh the xenografts.

### 2.10. Survival Analyses and Statistics

Overall survival (OS) and disease-specific survival (DSS) analyses were performed using the Kaplan–Meier methods, stratified by SMPD3 expression levels and other clinicopathological factors. Survival curves were compared using the log-rank test. Multivariate survival analysis was conducted using Cox regression models to identify independent prognostic factors. Hazard ratios (HRs) and 95% confidence intervals (CIs) were calculated for each variable. Stratified survival analyses were carried out to investigate the impact of SMPD3 expression on different patient subgroups. Survival analyses of TCGA HCC cohort were conducted via the online platform [25]. Statistical analyses were performed using SPSS22.0 software. Categorical variables were assessed with the chi-square test. A *p* value less than 0.05 was considered statistically significant and was emphasized with the symbol ∗. We used three mice in each group for the animal validation experiments. For the cellular experiments, three biological replicates were conducted.

### 2.11. Ethics

The study was conducted in accordance with the principles outlined in the Declaration of Helsinki and approved by the institutional review board. All human research participants provided informed consent for their participation in the study and the use of their clinical data. Data from TCGA was used in compliance with data access guidelines and policies. The animal experiments conformed to ethical guidelines and were approved by the Medical Experimental Animal Care Commission of Harbin Medical University Cancer Hospital.

## 3. Result

### 3.1. Differential Expression of SMPD3 in Normal Liver Tissues and HCC

**Figure 1:**
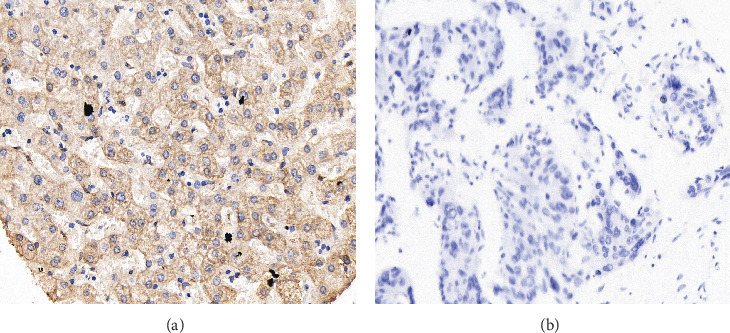
Immunohistochemical staining. Representative (a) high SMPD3 expression level in normal liver tissues and (b) low SMPD3 expression in HCC tissues as revealed by immunohistochemical staining.

IHC staining was conducted to evaluate SMPD3 protein expression levels in normal liver tissues and HCC tissues. Representative images of SMPD3 staining are shown in Figure 1. In normal liver tissues (Figure 1(a)), high SMPD3 expression level was observed, indicated by intense cytoplasmic staining in hepatocytes. Conversely, HCC tissues (Figure 1(b)) exhibited a notable decrease in SMPD3 protein expression, with weaker staining intensity compared to normal liver tissues. This differential expression pattern suggests a potential dysregulation of SMPD3 in HCC, implicating its involvement in HCC pathogenesis and progression.

### 3.2. Correlations Between SMPD3 Expression and Patients' Characteristics

Table 1 summarizes the relationships between SMPD3 expression levels and various clinical and pathological characteristics in a cohort of 109 HCC patients from our hospital. The cohort was divided into groups based on low and high levels of SMPD3 expression in tumor tissues, according to the median IHC score. No significant correlations were observed between SMPD3 expression levels and the age, sex, HBV infection status, AFP levels, pathological grade, tumor differentiation, or resection margin status of the patients. This suggests that SMPD3 expression does not appear to be associated with these particular clinical or pathological factors in this cohort.

However, a significant relationship was observed between SMPD3 expression and tumor size (*p* < 0.001). Tumors measuring less than 5.0 cm in size were more likely to exhibit high SMPD3 expression level, whereas larger tumors (≥ 5.0 cm) were more likely to have low SMPD3 expression level. Similarly, there was a statistically significant association between SMPD3 expression and portal vein invasion (*p* = 0.016). Tumors without portal vein invasion were more likely to express high levels of SMPD3, while those with invasion tended to have lower SMPD3 expression. The TNM stage of the tumor also showed a significant correlation with SMPD3 expression levels (*p* = 0.016). Early-stage tumors (TNM I–II) were more likely to exhibit high SMPD3 expression level compared to late-stage tumors (TNM III–IV), which were more likely to have low SMPD3 expression level.

These findings suggest that higher SMPD3 expression may be linked with more favorable tumor characteristics, such as smaller size, lack of portal vein invasion, and earlier TNM stage, therefore supporting the potential role of SMPD3 as a beneficial factor in HCC progression and underscoring the need for further investigation into its clinical implications in HCC.

### 3.3. Survival Analyses of Our Cohort

**Figure 2:**
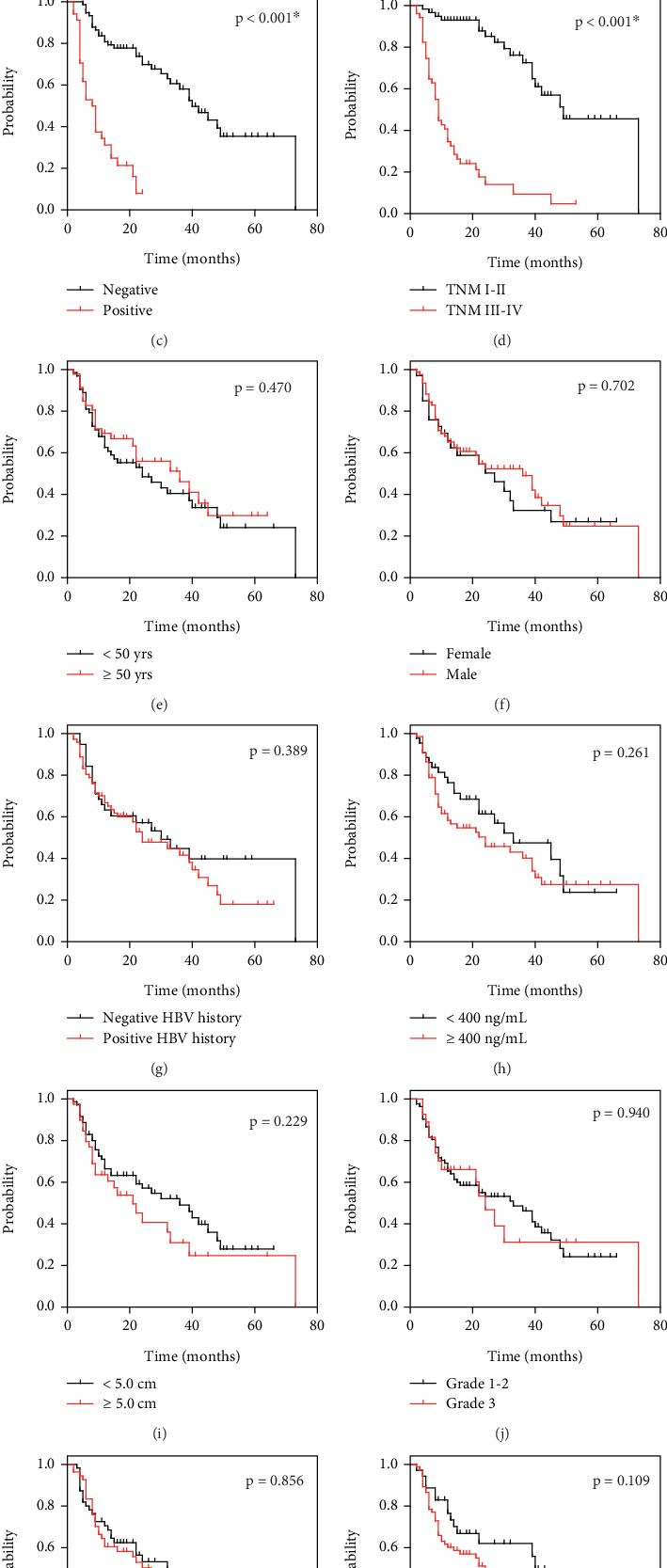
The Kaplan–Meier survival curves assessing prognosis in HCC cases. Overall survival curves were established for (a) all HCC cases and stratified by (b) SMPD3 expression level, (c) portal vein invasion, (d) TNM stage, (e) age, (f) sex, (g) HBV infection history, (h) serum AFP level, (i) tumor size, (j) pathological grade, (k) differentiation, and (l) resection margin. Curves were compared using the log-rank test.

Univariate survival analyses were conducted using the Kaplan–Meier analyses to evaluate the impact of various clinical and pathological variables on OS among 109 HCC patients (Table 2). The Kaplan–Meier survival curves were established to evaluate the prognosis of HCC patients based on a range of clinical and pathological characteristics (Figure 2(a)). In detail, patients were stratified based on several variables, including age, sex, HBV infection status, AFP level, tumor size, pathological grade, differentiation, resection margin, portal vein invasion, TNM stage, and SMPD3 expression level.

Among the analyzed factors, several were found to be significantly associated with OS. Notably, high SMPD3 expression level was strongly correlated with longer OS (44.190 ± 4.288 months) compared to patients with low SMPD3 expression level (20.449 ± 2.550 months) (Figure 2(b), *p* < 0.001). Patients with negative portal vein invasion also experienced significantly longer OS (43.334 ± 3.569 months) compared to those with positive portal vein invasion (10.347 ± 1.263 months) (Figure 2(c), *p* < 0.001). TNM stage also showed a significant association with OS. Patients with early-stage tumors (TNM I–II) had a considerably longer OS (51.252 ± 3.981 months) compared to those with late-stage tumors (TNM III–IV) (14.324 ± 2.060 months) (Figure 2(d), *p* < 0.001). Other variables such as age, sex, HBV infection, AFP level, tumor size, pathological grade, differentiation, and resection margin did not demonstrate significant associations with OS in this cohort (Figures 2(e), 2(f), 2(g), 2(h), 2(i), 2(j), 2(k), and 2(l), *p* > 0.05).

Overall, these univariate analyses indicate that SMPD3 expression, portal vein invasion, and TNM stage are key prognostic factors affecting OS in HCC patients. Multivariate Cox regression analysis was then performed to identify independent prognostic factors affecting OS in HCC patients.  Table 3 presents the HRs and 95% CIs for key variables. The analysis confirmed that TNM stage was a strong independent predictor of survival. Patients with advanced TNM stages (III–IV) had a significantly higher HR of 5.902 (95% CI: 2.880–12.098, *p* < 0.001) compared to those with early stages (I–II), indicating a substantially worse prognosis. High SMPD3 expression level was associated with a significantly reduced HR of 0.494 (95% CI: 0.260–0.938, *p* = 0.031) compared to low SMPD3 expression level. This finding reinforces the beneficial role of SMPD3 in HCC outcomes and suggests its potential as a prognostic marker. In contrast, while positive portal vein invasion had an elevated HR of 1.608 (95% CI: 0.785–3.294), it did not reach statistical significance (*p* = 0.194), suggesting that its impact on survival may be less pronounced when other variables are taken into account.

In summary, the multivariate analysis highlights TNM stage and SMPD3 expression level as significant independent predictors of OS in HCC patients, providing valuable insights for risk stratification and therapeutic decision-making.

### 3.4. Survival Analysis of TCGA HCC Cohort

**Figure 3:**
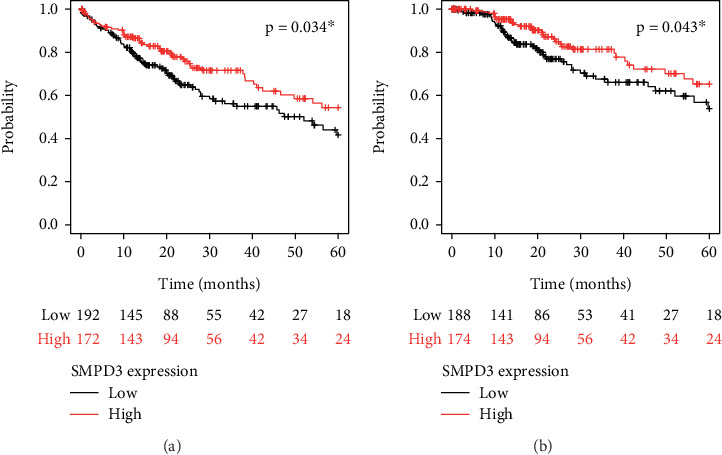
The Kaplan–Meier survival curves evaluating the prognostic role of SMPD3 in TCGA HCC cohort. Survival analysis for HCC cases in TCGA cohort was performed according to SMPD3 expression levels for (a) overall survival and (b) disease-specific survival.

The Kaplan–Meier survival curves were constructed to evaluate the prognostic role of SMPD3 expression in TCGA HCC cohort. The analyses assessed OS and DSS based on different levels of SMPD3 expression, showing a clear separation between survival curves for patients with high versus low SMPD3 expression levels. Patients with high SMPD3 expression level had significantly longer survival compared to those with low expression level (Figures 3(a) and 3(b)).

**Figure 4:**
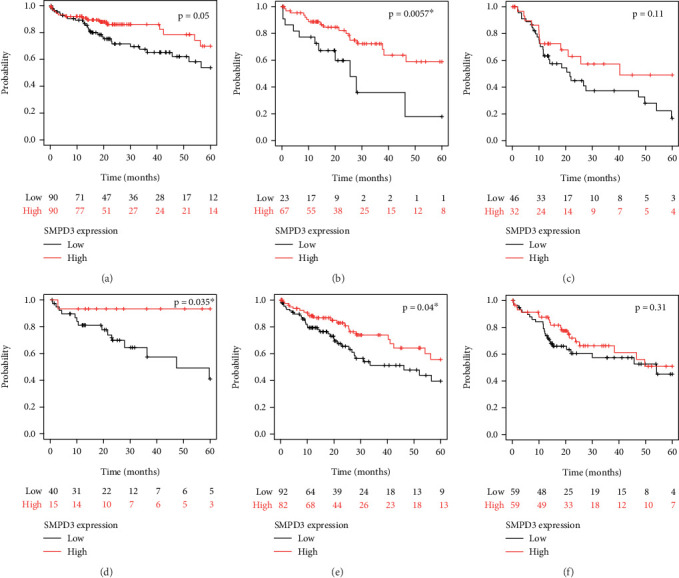
Subgroup overall survival analyses of HCC cases according to SMPD3 levels. (a–c) Prognostic impact of SMPD3 expression in HCC patients at Stages T1, T2, and T3, respectively. Results show borderline significance in T1 patients (*p* = 0.05), significant differences in T2 patients (*p* = 0.00057), and no significance in T3 patients (*p* = 0.11). (d–f) Prognostic impact of SMPD3 expression in HCC patients with Pathological Grades 1, 2, and 3, respectively. Grade 1 and Grade 2 patients showed significant differences (*p* = 0.035 and *p* = 0.04), while Grade 3 showed no statistical significance (*p* = 0.31).

Stratified OS analyses were conducted in HCC cases from TCGA cohort to evaluate the prognostic impact of SMPD3 expression across different clinical and pathological subgroups. The prognostic impact of SMPD3 expression was assessed in HCC patients based on different tumor stages (T1, T2, and T3) (Figures 4(a), 4(b), and 4(c)). Patients with Stage T1 tumors showed a trend toward longer survival with high SMPD3 expression level (*p* = 0.05), although it was of borderline statistical significance. In Stage T2 patients, high SMPD3 expression level was associated with significantly longer survival (*p* = 0.00057), indicating a strong protective effect. However, in Stage T3 patients, there was no statistically significant difference in survival outcomes based on SMPD3 expression (*p* = 0.11). Similar stratification analyses were conducted based on pathological grade (1, 2, and 3) (Figures 4(d), 4(e), and 4(f)). In Grade 1 and Grade 2 patients, high SMPD3 expression level correlated with significantly better survival (*p* = 0.035 and *p* = 0.04, respectively). In contrast, Grade 3 patients did not demonstrate a significant difference in survival outcomes based on SMPD3 expression (*p* = 0.31).

**Figure 5:**
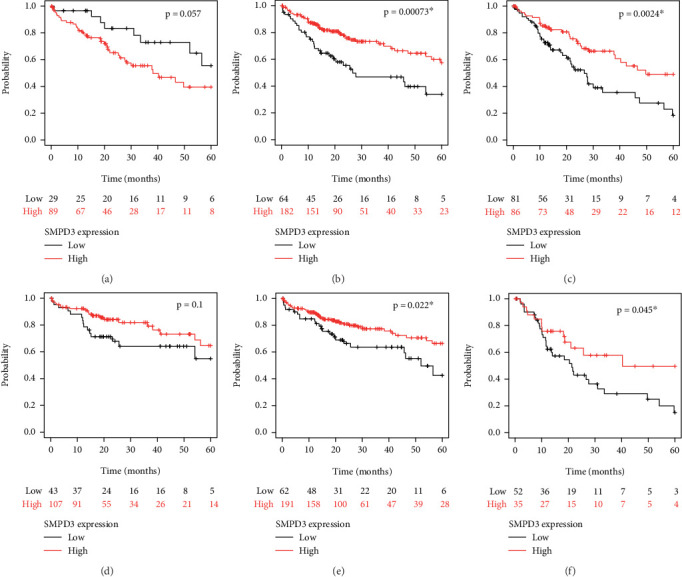
Subgroup overall survival analyses of HCC cases according to SMPD3 levels. (a, b) Female patients did not exhibit significant differences in survival between high and low SMPD3 groups (*p* = 0.057), whereas male patients demonstrated significantly worse survival in the high SMPD3 group (*p* < 0.001). (c, d) Patients with a negative HBV infection history showed significant differences in survival between high and low SMPD3 groups (*p* = 0.002), while patients with a positive HBV infection history did not (*p* = 0.1). (e, f) SMPD3 expression had a significant impact on HCC survival for patients with TNM Stages I–II (*p* = 0.022) and TNM Stages III–IV (*p* = 0.045).

In addition, we assessed the survival differences between high and low SMPD3 expression levels in female and male patients (Figures 5(a) and 5(b)). In female patients, there was a trend toward better survival with high SMPD3 expression level, though it did not reach statistical significance (*p* = 0.057). In male patients, high SMPD3 expression level was associated with significantly worse survival compared to low SMPD3 expression level (*p* < 0.001). Survival differences based on SMPD3 expression level were also examined in patients with negative and positive HBV infection history (Figures 5(c) and 5(d)). Patients without an HBV infection history showed a significant survival benefit with high SMPD3 expression level (*p* = 0.002). However, for patients with a positive HBV infection history, no significant survival differences were observed based on SMPD3 expression (*p* = 0.1). The impact of SMPD3 expression on survival was finally assessed in patients with early (TNM Stages I–II) and advanced (TNM Stages III–IV) disease stages (Figures 5(e) and 5(f)). In both stages, high SMPD3 expression level was associated with significantly better survival compared to low SMPD3 expression level (*p* = 0.022 and *p* = 0.045, respectively).

**Figure 6:**
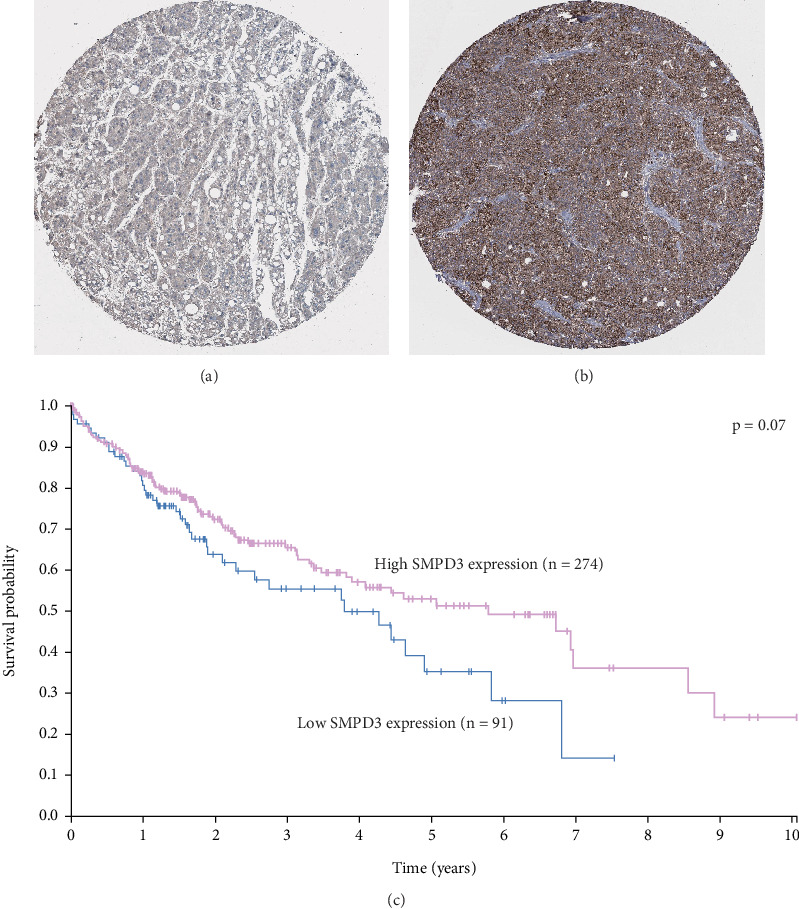
Immunohistochemical staining and survival of the HCC cohort from the Human Protein Atlas database. Representative (a) negative and (b) positive SMPD3 expression in HCC tissues as revealed by immunohistochemical staining. (c) The overall survival curve in the CC cohort from the Human Protein Atlas database showed that patients with lower SMPD3 protein expression exhibited worse survival; however, the statistical difference was not significant (*p* = 0.07).

To further validate our findings, we utilized the Human Protein Atlas database on SMPD3 expression in HCC tissues. Similar to our cohort, the HCC cohort in the Human Protein Atlas database also showed diverse SMPD3 expression levels (Figures 6(a) and 6(b)). Importantly, although the *p* value did not reach statistical significance, it indeed showed a trend that low SMPD3 was correlated with worse survival (Figure 6(c), *p* = 0.07) in the HCC cohort in the Human Protein Atlas database.

### 3.5. Expression and Antitumor Effect of SMPD3 in HCC Cell Lines

**Figure 7:**
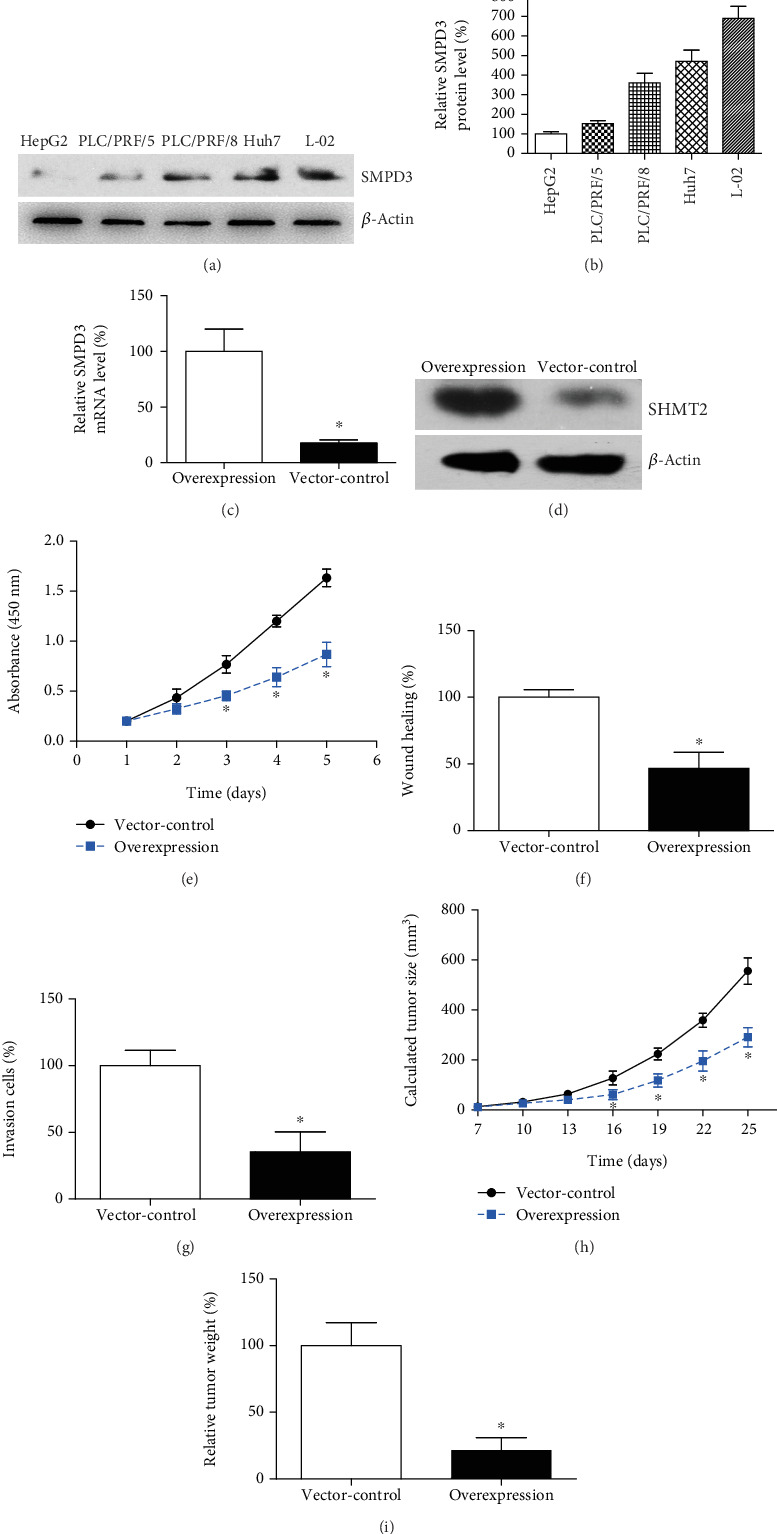
Overexpression of SMPD3 inhibits HCC progression both in vitro and in vivo. (a, b) Western blot and semiquantitative analysis showed lower SMPD3 protein levels in HCC cell lines compared to L02 hepatocyte cells. (c) RT-qPCR confirmed significantly increased mRNA levels of SMPD3 in PLC/PRF/8 cells transfected with SMPD3-plasmid compared to vector-control cells. (d) Western blotting validated efficient SMPD3 overexpression in PLC/PRF/8 cells. (e) CCK8 assay indicated significantly reduced proliferation in SMPD3-overexpressing PLC/PRF/8 cells compared to vector-control cells. (f) According to the wound-healing assay, PLC/PRF/8 cells transfected with SMPD3 overexpression exhibited impaired migration capacity compared to those transfected with vector plasmids. (g) Matrigel transwell experiments revealed that the invasion process of PLC/PRF/8 cells was also inhibited after overexpressing SMPD3. (h) Nude mice were subcutaneously injected with SMPD3-overexpression PLC/PRF/8 cells or vector-control PLC/PRF/8 cells, respectively. Then, the growth of xenografts was monitored and plotted, which showed significantly slower growth of SMPD3-overexpression xenografts. (i) The isolated mouse xenografts were weighted, indicating that overexpressing SMPD3 resulted in a smaller xenograft size.

Protein expression of SMPD3 was evaluated in a human nontumorous hepatocyte cell line (L02) and four human HCC cell lines (HepG2, PLC/PRF/5, PLC/PRF/8, and Huh7) using Western blot analysis (Figure 7(a)). The results showed lower SMPD3 protein expression in all four HCC cell lines compared to L02 cells, with the lowest level observed in HepG2 cells (Figure 7(b)). To investigate the antitumor effect of SMPD3, we conducted an SMPD3-overexpression assay using PLC/PRF/8 cells, which exhibited moderate SMPD3 levels. After 72 h of transfection, RT-qPCR (Figure 7(c)) and Western blotting (Figure 7(d)) confirmed a significant increase in SMPD3 expression in SMPD3-overexpression cells compared to vector-control cells, indicating successful overexpression.

We assessed tumor-related phenotypes in SMPD3-overexpression and control cells, including proliferation, migration, and invasion capacities. The CCK8 assay demonstrated a significant decrease in the proliferation rate of SMPD3-overexpression cells compared to vector-control cells (Figure 7(e)). Wound-healing experiments revealed a marked reduction in migration in SMPD3-overexpression cells (Figure 7(f)). Additionally, the Matrigel transwell assay showed that SMPD3 overexpression significantly impeded the invasion of HCC cells (Figure 7(g)), aligning with our clinical observations that SMPD3 expression is inversely associated with advanced TNM stage.

### 3.6. Overexpression of SMPD3 Inhibits HCC Growth in Nude Mice

Given the impact of SMPD3 on HCC growth in vitro, we aimed to verify our findings in vivo. A mouse model was established by subcutaneously injecting transfected PLC/PRF/8 cells into nude mice (*n* = 3 in each group) and monitoring xenograft growth (Figure 7(h)). Xenografts derived from SMPD3-overexpression cells exhibited significantly slower growth rates compared to those from scramble cells. At the designated endpoint, xenografts from SMPD3-overexpression cells had lighter weights than those from the control group (Figure 7(i)). These findings confirm the antitumor effect of SMPD3 in vivo, supporting its potential as a therapeutic target in HCC.

## 4. Discussions

The present study provides in-depth insights into the antitumor role of SMPD3 in HCC, which may have significant clinical implications. High SMPD3 expression level was associated with improved OS and DSS in our hospital cohort and TCGA cohort. These findings align with previous research suggesting a potential tumor-suppressive function of SMPD3 in various cancers [26–29].

In vitro experiments demonstrated that SMPD3 overexpression in HCC cell lines significantly reduced cell proliferation, migration, and invasion, while in vivo experiments in nude mice showed that SMPD3 overexpression inhibited tumor growth. Our data is consistent with a previous study reporting the antiproliferation role of SMPD3 in HCC cell line [30] and can also regulate tumor cell migration [31, 32]. These antitumor effects may be linked to the modulation of various signaling pathways, including those involved in cell cycle regulation and apoptosis.

The enzymatic activity of SMPD3 results in the hydrolysis of sphingomyelin to produce ceramide, a bioactive lipid that has been implicated in tumor suppression and cellular stress responses. Ceramide generated by SMPD3 can induce apoptosis through various mechanisms. It acts as a second messenger in stress signaling pathways, leading to the activation of caspases, which are proteases essential for the execution of apoptosis. Ceramide has been shown to interact with mitochondria, promoting the release of cytochrome c and the subsequent activation of the apoptotic cascade. SMPD3-mediated ceramide production has been linked to the inhibition of cell proliferation. Ceramide can suppress the Akt pathway, a critical regulator of cell survival and growth. By inhibiting Akt phosphorylation, ceramide reduces the downstream signaling that promotes cell cycle progression and proliferation. Additionally, ceramide can enhance the expression of cell cycle inhibitors such as p21 and p27, further halting cell cycle progression.

Ceramide produced by SMPD3 is involved in cellular responses to various stress stimuli, including oxidative stress and chemotherapeutic agents. It has been reported that ceramide can sensitize cancer cells to apoptosis induced by these stresses, potentially enhancing the efficacy of anticancer treatments. Recent studies have suggested that SMPD3 and ceramide play a role in the regulation of autophagy, a process that can promote cell survival or death depending on the context. Ceramide can trigger autophagic cell death in cancer cells by modulating the activity of autophagy-related proteins such as Beclin-1 and Atg5. This mechanism provides another pathway through which SMPD3 exerts its tumor-suppressive effects. SMPD3 and ceramide also influence the tumor microenvironment. Ceramide can modulate the behavior of various cell types within the tumor stroma, including immune cells and endothelial cells. For example, ceramide has been shown to enhance the antitumor activity of natural killer (NK) cells and promote the normalization of tumor blood vessels, which can improve the delivery of chemotherapeutic agents and reduce metastasis.

The antitumor effects of SMPD3 may also involve cross-talk with other signaling pathways. For instance, ceramide can interact with the mitogen-activated protein kinase/extracellular signal-regulated kinase (MAPK/ERK) pathway, influencing cellular outcomes such as differentiation and apoptosis. Additionally, the interplay between ceramide and other sphingolipids, like sphingosine-1-phosphate (S1P), which has prosurvival properties, adds another layer of complexity to the regulation of tumor progression by SMPD3. Taken together, the mechanistic insights into SMPD3's role in HCC highlight its multifaceted impact on tumor biology. By regulating key processes such as apoptosis, cell proliferation, stress responses, autophagy, and the tumor microenvironment, SMPD3 emerges as a significant tumor suppressor. Further research into these mechanisms will enhance our understanding of SMPD3's potential as a therapeutic target and may lead to the development of novel treatment strategies for HCC.

Our study also suggests potential differences in the impact of SMPD3 expression across patient subgroups. For instance, high SMPD3 expression was beneficial for patients with a negative HBV infection history, while it was not significantly impactful for those with a positive HBV infection history. These findings highlight the complexity of HCC and the need for personalized approaches in its management. Similarly, the observation that SMPD3 expression led to significantly worse survival in male patients compared to females underscores the necessity of investigating sex-specific responses in future research.

Comparing our results with existing literature, previous studies in other cancer types have shown SMPD3 to have tumor-suppressive properties, such as in colorectal and breast cancers. These findings suggest that SMPD3 may exert a broader antitumor effect across various malignancies. Understanding its mechanism of action could reveal novel therapeutic targets for HCC and other cancers. Furthermore, some studies have suggested that SMPD3 may impact key signaling pathways, such as the MAPK/ERK pathway, which are known to play a crucial role in tumor growth and metastasis. Elucidating the interactions between SMPD3 and these pathways could provide insights into how SMPD3 influences tumor progression and patient outcomes. Meanwhile, recent studies implicated that SMPD3 may help modulate immunotherapeutic efficiency in various cancers [33, 34].

Our study, while providing significant insights into SMPD3's role in HCC, has several limitations. The retrospective nature of part of the study inherently carries selection bias and cannot establish causality. Our sample size, though adequate, may be limited, and the study population primarily represents a single geographical region, potentially affecting the generalizability of our findings across diverse populations. HCC exhibits considerable heterogeneity due to variations in risk factors such as Hepatitis B and C prevalence, aflatoxin exposure, and lifestyle factors, necessitating validation in more diverse cohorts. Functional validation through in vitro and in vivo studies is also needed to corroborate our findings and confirm SMPD3's biological relevance in HCC. Potential confounding factors, such as coexisting medical conditions, treatment variations, and lifestyle factors, were not fully accounted for, requiring future prospective studies with comprehensive data collection. Methodologically, while we used IHC for protein expression analysis, it can be variable; thus, quantitative methods like Western blotting or mass spectrometry could complement our data. Additionally, the use of single-gene analysis may overlook complex interactions involved in HCC pathogenesis. Addressing these limitations provides a balanced perspective, highlighting areas for future research and the need for larger, more diverse, and prospective studies to confirm our results and fully elucidate SMPD3's role in HCC.

In conclusion, our study adds to the growing body of evidence supporting the antitumor role of SMPD3 in HCC. High SMPD3 expression level was associated with improved survival outcomes, suggesting its potential utility as a prognostic marker and therapeutic target, for example, by targeting exosome release [35]. Continued research is necessary to further explore SMPD3's mechanism of action and its clinical applications in HCC management. By targeting SMPD3 or its downstream effects, novel treatment strategies for HCC may emerge, potentially leading to improved patient outcomes and survival rates.

## Figures and Tables

**Table 1:** Expression of SMPD3 in HCC patients.

**Variables**	**Cases**	**SMPD3 level**		**p** ** value**
	**(** **n** = 109**)**	**Low (** **n** = 55**)**	**High (** **n** = 54**)**	
Age				
< 50 years	63	35	28	0.213
≥ 50 years	46	20	26	
Sex				
Female	33	16	17	0.786
Male	76	39	37	
HBV infection				
Negative	38	16	22	0.202
Positive	71	39	32	
AFP level				
< 400 ng/mL	43	19	24	0.290
≥ 400 ng/mL	66	36	30	
Tumor size				
< 5.0 cm	70	24	46	< 0.001⁣^∗^
≥ 5.0 cm	39	31	8	
Pathological grade				
Grades 1–2	82	39	43	0.292
Grade 3	27	16	11	
Differentiation				
Well/moderate	55	31	24	0.213
Poor/undifferentiated	54	24	30	
Resection margin				
≥ 1.0 cm	35	18	17	0.889
< 1.0 cm	74	37	37	
Portal vein invasion				
Negative	75	32	43	0.016⁣^∗^
Positive	34	23	11	
TNM stage				
TNM I–II	58	23	35	0.016⁣^∗^
TNM III–IV	51	32	19	

⁣^∗^Statistically significant by Pearson's chi-square test.

**Table 2:** Univariate overall survival (OS) by Kaplan–Meier analyses.

**Variables**	**Patients (** **n** = 109**)**	**OS months (** **m** **e** **a** **n** ± **SD)**	**p** ** value**
Age			
< 50 years	63	32.563 ± 3.888	0.470
≥ 50 years	46	34.023 ± 3.993	
Sex			
Female	33	30.743 ± 4.595	0.702
Male	76	34.806 ± 3.697	
HBV infection			
Negative	38	38.602 ± 5.273	0.389
Positive	71	30.242 ± 3.127	
AFP level			
< 400 ng/mL	43	35.291 ± 4.117	0.261
≥ 400 ng/mL	66	32.477 ± 3.862	
Tumor size			
< 5.0 cm	70	34.570 ± 3.248	0.229
≥ 5.0 cm	39	30.248 ± 5.136	
Pathological grade			
Grades 1–2	82	32.529 ± 2.963	0.940
Grade 3	27	33.776 ± 6.687	
Differentiation			
Well/moderate	55	32.262 ± 3.547	0.856
Poor/undifferentiated	54	34.125 ± 4.283	
Resection margin			
≥ 1.0 cm	35	41.146 ± 5.462	0.109
< 1.0 cm	74	29.682 ± 3.141	
Portal vein invasion			
Negative	75	43.334 ± 3.569	< 0.001⁣^∗^
Positive	34	10.347 ± 1.263	
TNM stage			
TNM I–II	58	51.252 ± 3.981	< 0.001⁣^∗^
TNM III–IV	51	14.324 ± 2.060	
SMPD3 level			
Low	55	20.449 ± 2.550	< 0.001⁣^∗^
High	54	44.190 ± 4.288	

⁣^∗^Statistically significant by log-rank test.

**Table 3:** Multivariate analysis by Cox regression method.

**Variables**	**Hazard ratio**	**95% CI**	**p** ** value**
Portal vein invasion (positive vs. negative)	1.608	0.785–3.294	0.194
TNM stage (III/IV vs. I–II)	5.902	2.880–12.098	< 0.001⁣^∗^
SMPD3 level (high vs. low)	0.494	0.260–0.938	0.031⁣^∗^

⁣^∗^Statistically significant by Cox regression analysis.

## Data Availability

Data will be available upon reasonable request.
